# Electrocardiographic abnormalities in patients with microtia

**DOI:** 10.1038/s41598-024-60610-9

**Published:** 2024-05-03

**Authors:** Yang Yang, Xiaoying Tian, Pengfei Sun, Xiaoli Zhao, Jintian Hu, Bo Pan

**Affiliations:** Plastic Surgery Hospital, Chinese Academy of Medical Sciences, Peking Union Medical College, Beijing, 100144 China

**Keywords:** Electrocardiographic abnormalities, Microtia, Congenital heart defect, Occurrence, Paediatric research, Diagnosis, Orthopaedics

## Abstract

The main objective of this study was to investigate the incidence and characteristics of electrocardiographic abnormalities in patients with microtia, and to explore cardiac maldevelopment associated with microtia. This retrospective study analyzed a large cohort of microtia patients admitted to Plastic Surgery Hospital, Chinese Academy of Medical Sciences and Peking Union Medical College, from September 2017 to August 2022. The routine electrocardiographic reports of these patients were reviewed to assess the incidence and characteristics of abnormalities. The study included a total of 10,151 patients (5598 in the microtia group and 4553 in the control group) who were admitted to the Plastic Surgery Hospital of Peking Union Medical College. The microtia group had a significantly higher incidence of abnormal electrocardiographies compared to the control group (18.3% vs. 13.6%, *P* < 0.01), even when excluding sinus irregularity (6.1% vs. 4.4%, *P* < 0.01). Among the 1025 cases of abnormal electrocardiographies in the microtia group, 686 cases were reported with simple sinus irregularity. After excluding sinus irregularity as abnormal, the most prevalent abnormalities was right bundle branch block (37.5%), followed by sinus bradycardia (17.4%), ST-T wave abnormalities (13.3%), atrial rhythm (9.1%), sinus tachycardia (8.3%), and ventricular high voltage (4.7%). Less common ECG abnormalities included atrial tachycardia (2.1%), ventricular premature contraction (2.4%), and ectopic atrial rhythm (1.8%). atrioventricular block and junctional rhythm were present in 1.2% and 0.9% of the cases, respectively. Wolff Parkinson White syndrome and dextrocardia had a lower prevalence, at 0.6% and 0.9%, respectively. The occurrence of electrocardiographic abnormalities in microtia patients was found to be higher compared to the control group. These findings highlight the potential congenital defect in cardiac electrophysiology beyond the presence of congenital heart defect that coincide with microtia.

Microtia is a congenital malformation, affecting approximately 1 in 6,000 to 1 in 12,000 live births worldwide^1,2^. This condition manifests as the underdevelopment or absence of one or both external ears and is often accompanied by varying degrees of hearing impairment. Historically, the primary focus of research and clinical care for microtia patients has been on addressing the craniofacial aspects of the condition, including surgical reconstruction of the external ear and management of hearing loss^3,4^.

However, recent studies have brought attention to the potential systemic manifestations of microtia, sparking interest in exploring the connections between this craniofacial anomaly and other health concerns^3,5–8^. One such area of investigation is the occurrence of congenital heart diseases (CHD) (^8–12^ in microtia patients, which has been reported in several clinical studies.

While, heart development abnormalities in individuals extend beyond structural defects and encompasses electrophysiological irregularities, and these electrophysiological defects can lead to a spectrum of cardiac issues, including arrhythmias and conduction abnormalities. Additionally, individuals suffering from various inborn heart anomalies are predisposed to arrhythmias attributed to their underlying structural cardiac condition and the anomalous anatomical conduction system and conduction tissue linked to the structural malfunction^13^. Therefore, Electrocardiography (ECG), as the basic diagnostic tool used to record the electrical activity of the heart, is essential in identifying various cardiac conditions and has profound clinical implications.

This comprehensive study aims to bridge the knowledge gap by conducting a thorough examination of ECG findings in a large cohort of microtia patients. By systematically analyzing ECG data, assessing the clinical implications of these abnormalities.

## Methods

### Study population

Data from September 2017 to August 2022 were retrospectively collected. The inclusion criteria were (I) patients at admission to Plastic Surgery Hospital of Peking Union Medical College, Beijing, China; (II) patients diagnosed as congenital microita into microtia group; (III) patients diagnosed with skin diseases (pigmented nevus, capillary hemangioma, et al.) or acquired injury (burn injury, scar, et al.) into control group, excluding the likelihood of heart involvement in other structural malformation disease ; (IV) and patients aged between 0 and 12 years old diminishing the likelihood of acquired heart diseases. Exclusion criteria were (I) patients without routine ECG reports at admission (due to personal issues, e.g. respiratory tract infection or refusing treatments); and (II) control group patients that co-diagnosed with congenital structural defect except heart.

### ECG evaluation and further examination

ECGs were obtained using ECG machine (CardiMax FX-8600, FUKUDA, Beijing, China) with standard lead placements, and each recording lasted for a minimum of five minutes to ensure adequate data capture. The ECGs were recorded at rest, and any specific circumstances that might affect cardiac electrical activity (e.g., medications, recent surgeries) were documented.

ECG recordings were initially reviewed by trained cardiac technicians for any immediate abnormalities or artifacts. Subsequently, the ECGs were interpreted by experienced clinicians who were blinded to the patients' clinical information and microtia status. The evaluation included assessment of rhythm, heart rate, PR interval, QRS duration, QT interval, and the presence of any arrhythmias or conduction abnormalities, such as atrioventricular block or bundle branch block.

‘Normal ECG’ or ‘Roughly normal ECG’ in reports were considered as normal {ECG ( −)}, and others were considered as abnormal {ECG ( +)}.

Microtia patients with identified ECG abnormalities or clinical concerns underwent further cardiac evaluation, which may have included echocardiography (GE LOGIQ E11, California, USA) or additional imaging studies, as deemed necessary based on the initial ECG findings.

### Statistical analysis

Continuous variables are presented as the mean ± standard deviation and quartile. The categorical variables are presented as numbers and percentage, compared using the statistical program Chi-square test or Fisher’s exact test as appropriate. All results with *P* < 0.05 were considered statistically significant. The aforementioned analyses were performed using SPSS Statistic, version 26.0 software (IBM Corp., Armonk, NY, USA, www.ibm.com/products/spss-statistics), and GraphPad Prism, version 10.0.2 software (GraphPad Software, Boston, MA, USA, www.graphpad.com).

### Ethical approval

All procedures in this study were conducted in accordance with the Institutional Research Ethics Board of Plastic Surgery Hospital, Chinese Academy of Medical Sciences, Peking Union Medical College (No. 2023-184) approved protocols.

### Informed consent

Individual consent for this retrospective analysis was waived by Plastic Surgery Hospital, Chinese Academy of Medical Sciences, Peking Union Medical College.

## Results

From September 2017 to August 2022, 10,225 patients at admission to Plastic Surgery Hospital of Peking Union Medical College were compliance with inclusion critiria; 10,151 patients were included in the present study and 74 patients were excluded (23 patients without routine ECG reports at admission, 51 patients in the control group co-diagnosed with other congenital structural defect except heart). Of 10,151 patients included in the present study, the microtia group included 5598 patients and the control group included 4553 patients, with a mean age of 8.2 ± 2.9 years old and 6.1 ± 3.6 years old separately. And More detailed clinical characteristics of both groups are listed in Table 1.

**Table 1:** Demographic characteristics of the total patients.

Characteristic	Microtia group	Control group
No. of patients	5598	4553
Age, years		
Mean ± SD	8.2 ± 2.9	6.1 ± 3.6
Quartile	6, 9	4, 8
Gender, n(%)		
Male	4102 (73.3)	2234 (49.1)
Female	1496 (26.7)	2319 (50.9)

*SD* standard deviation.

**Figure 1:**
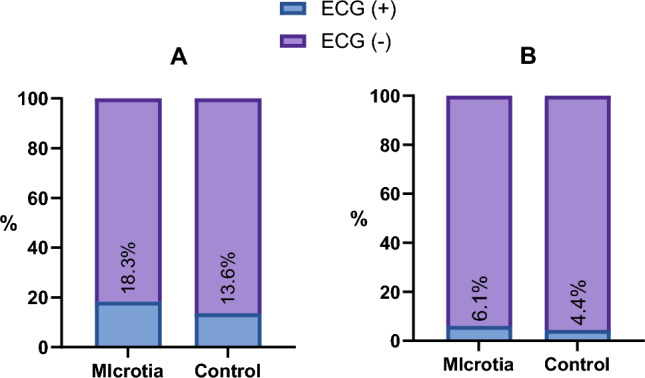
Percent bar graph about incidence of abnormal ECG in microtia and control group. (**A**) Sinus irregularity included in ECG ( +). (**B**) Sinus irregularity excluded in ECG ( +).

A significantly higher incidence of abnormal ECG was observed in microtia group (microtia vs. control: 18.3% vs. 13.6%, *P* < 0.01). Considering sinus irregularity common and generally benign variant of sinus irregularity with limited clinical significance, it was further specifically excluded from ECG abnormalities and subjected to re-analysis. Without sinus irregularity, the incidence of abnormal ECG was still higher in microtia group than control group with statistic difference (microtia vs. control: 6.1% vs. 4.4%, *P* < 0.01) (see Table 2 and Fig. 1).

**Table 2:** Comparisons of the incidence of abnormal ECG.

Variable	Microtia group	Control group	*P* value
ECG ( +), n(%)	1025 (18.3)	620 (13.6)	< 0.001
ECG ( +) excluding sinus arrhythmia, n(%)	339 (6.1)	201 (4.4)	< 0.001
Sinus irregularity isolated, n(%)	686 (12.2)	419 (9.2)	< 0.001

**Figure 2:**
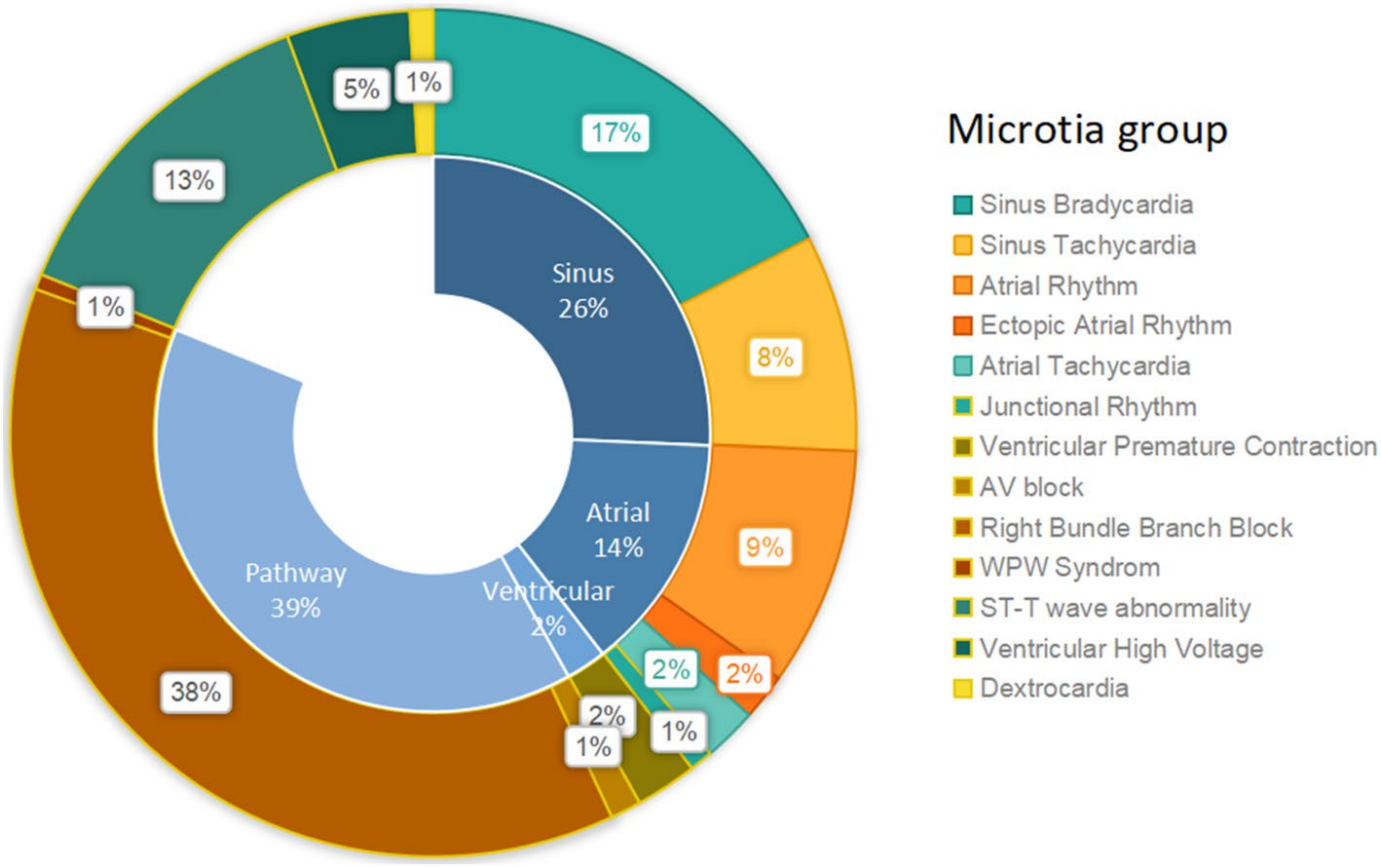
Double circle diagram about occurrence of forms in abnormal ECG in microtia group. Inner circle represents general classification of ECG abnormalities, and outer circle represents specific/subgrouped forms of ECG abnormalities. (sinus arrhythmia excluded). *AV block* atrioventricular block; *WPW syndrome* Wolff–Parkinson–White syndrome.

In 1025 cases of abnormal ECG in microtia group, 686 cases were reported with simple sinus irregularity. After excluding sinus irregularity as abnormal, the most prevalent abnormalities observed in this microtia patient group was right bundle branch block (RBBB), which was present in 37.5% of the cases. This was followed by sinus bradycardia (17.4%) and ST-T wave abnormalities (13.3%). While, Wolff–Parkinson–White syndrome (WPW Syndrome) and dextrocardia had lowest prevalence, at 0.6% and 0.9%, respectively. And detailed forms of ECG abnormalities were seen in Table 3 and Fig. 2.

**Table 3:** Occurrence of specific forms in abnormal ECG in microtia group (sinus arrhythmia excluded).

Variable	N	CR (%)
Sinus Bradycardia	59	17.4
Sinus Tachycardia	28	8.3
Atrial Rhythm	31	9.1
Ectopic Atrial Rhythm	6	1.8
Atrial Tachycardia	7	2.1
Junctional Rhythm	3	0.9
Ventricular Premature Contraction	8	2.4
AV Block	4	1.2
Right bundle branch block	127	37.5
WPW Syndrome	2	0.6
ST-T wave abnormalities	45	13.3
Ventricular High Voltage	16	4.7
Dextrocardia	3	0.9

*AV block* atrioventricular block; *WPW syndrome* Wolff–Parkinson–White syndrome; *CR* constitutive ratio.

**Figure 3:**
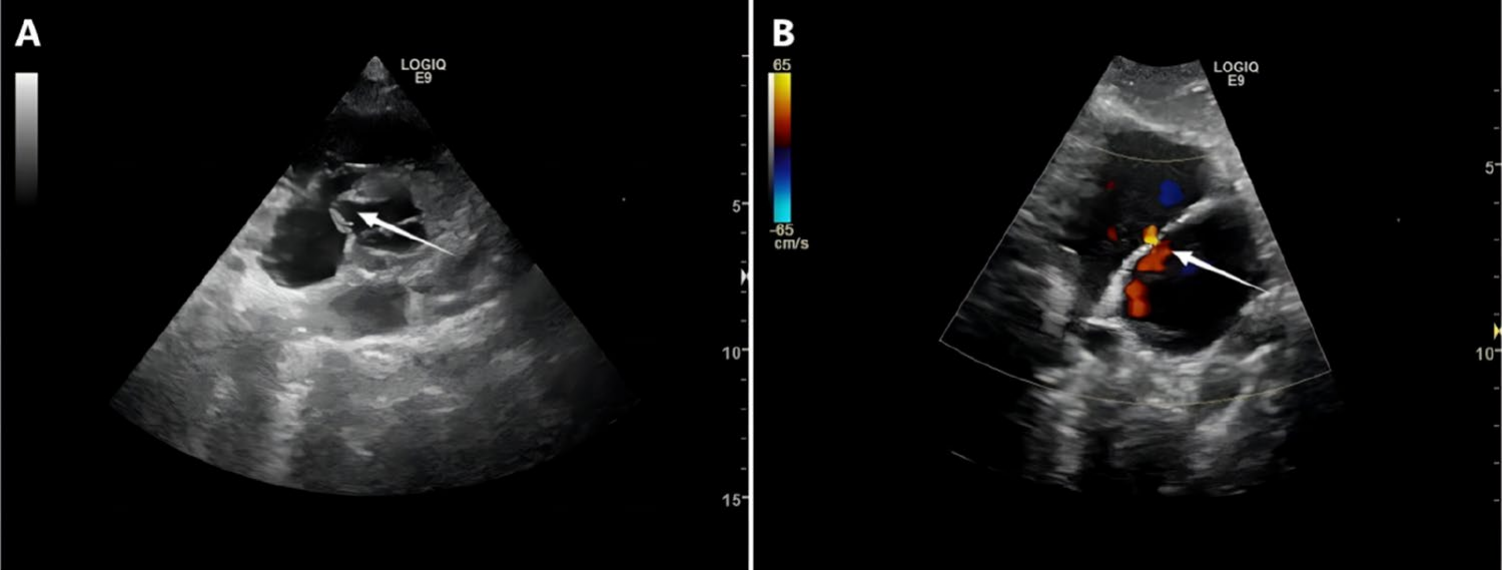
Cases of further echocardiography after abnormal ECG identified. (**A**) White arrow: Aortic sinus aneurysm. (**B**) White arrow: Atrial septal vdefect.

**Figure 4:**
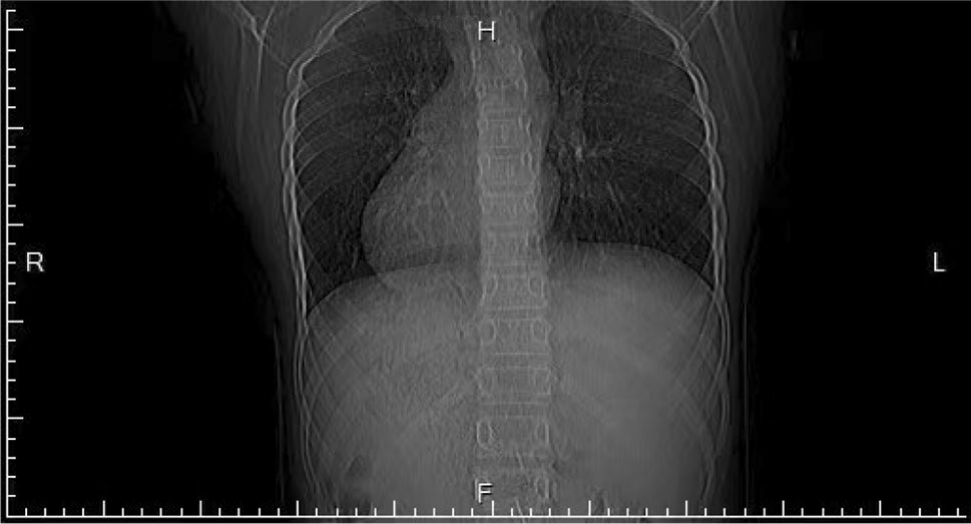
A case of dextrocardia with further X-ray after abnormal ECG identified.

The further examinations were done in some microtia patients with abnormal ECG. The typical cases were shown in Figs. 3 and 4.

## Discussion

The present study demonstrates a significantly higher prevalence of ECG abnormalities in the microtia patient group compared to a control group, even after excluding sinus arrhythmia. The elevated incidence of ECG abnormalities in microtia patients is of paramount clinical importance, as it points towards the presence of potential underlying cardiac issues in this unique patient population.

The recent body of research in microtia, a congenital anomaly ranking as the second most prevalent after cleft lip, has illuminated the intriguing possibility of systemic manifestations. One particular focus is the combined congenital heart diseases^8–12^. This study's comprehensive scope is underscored by the substantial size of the patient cohort, distinguishing it as a significant contribution to the understanding of microtia, its potential links to cardiac maldevelopment, and the concurrent occurrence of electrocardiographic (ECG) abnormalities. And our finding, the notably high incidence of ECG abnormalities in microtia patients, marked a noteworthy advancement in the understanding of this phenomenon.

To ensure the clinical relevance of our findings, we took a cautious approach by excluding sinus arrhythmia, a common and typically benign variant of sinus irregularity, from our analysis. Remarkably, even after this exclusion, the microtia group maintained a significantly higher incidence of ECG abnormalities compared to the control group. Besides, the incidence of sinus irregularity also showed statistic difference (12.2% vs. 9.2%,*P* < 0.001). These robust results suggest that the observed increase in ECG abnormalities in microtia patients cannot be solely attributed to benign variations but is indicative of clinically relevant cardiac irregularities.

For microtia population, previous researches demonstrated common combined congenital heart structure diseases are atrial septal defect (ASD), ventricular septal defect (VSD), and patent ductus arteriosus (PDA)^9,10^. And associated ECG findings include atrial arrhythmia, AV block, incomplete right bundle branch block (RBBB), and ventricular hypertrophy/high voltage, in the context of pulmonary overcirculation and right heart volume overload, which can lead to right atrial and right ventricular enlargement^14–16^. And it is consistent with our results that RBBB and atrial rhythm made up the majority of ECG presentation after excluding sinus irregularity.

In addition, atrial and ventricular arrhythmia, accounted for 16% (14% + 2%) of ECG abnormalities in microtia group, may be may be linked to residual hemodynamic abnormalities^13,17^. These abnormalities can arise from a combination of intrinsic congenital defects and post-surgical corrections, hinting at a multifaceted interplay between the structural integrity of the heart and its electrophysiological function. Atrial arrhythmias, such as atrial fibrillation or atrial flutter, may be predisposed by alterations in the atrial anatomy due to the underlying congenital defects associated with microtia. Additionally, post-surgical corrections might introduce changes in blood flow patterns, contributing to the development of these arrhythmias. Similarly, the presence of ventricular arrhythmias raises considerations about the overall electrophysiological stability of the ventricles in microtia individuals. And the notable occurrence of ST-T wave changes, comprising 13% of ECG abnormalities, suggested the possibility of dilated or hypertrophic cardiomyopathy secondary to cardiac malformation^18^.

Further, there were more specific ECG presentations in microtia group. Wolff-Parkinson (WPW) syndrome, known to be associated with abnormal electrical conduction encountering accessory pathway called bundle of Kent, occurs commonly in levo transposition of the great arteries (L-TGA), Ebstein anomaly and AV septal defect^13^. And Dextrocardia just directly shows the rare congenital condition that the positioning of the heart apex on the opposite side of the body^19^.

Sinus arrhythmia, despite being generally regarded as a benign ECG finding in the general population^20^, merits specific consideration in the context of microtia patients. Even the excluded sinus irregularity exhibited a statistically significant difference compared to the control group, raising intriguing questions about the potential clinical implications of this phenomenon within the microtia cohort. Sinus tachyarrhythmias may arise from enhanced automaticity, triggered activity, or a reentry mechanism, whereas bradyarrhythmias are typically a result of the deficient generation of impulses or the impediment of conduction through the heart's specific conduction system, potentially reflecting abnormalities in cardiac anatomy or electrophysiology^21^. While, sinus node dysfunction associated sinus arrhythmia, can also be either intrinsic or acquired, as an early or late postoperative complication. The proposed pathophysiology involves progressive fibrosis of the sinoatrial tissue, either following surgery or due to the long-term hemodynamic compromise resulting from a congenital heart defect^22,23^.

The identification of ECG abnormalities within the microtia population unveils a fascinating nexus between auricular and cardiac development, surpassing the conventional focus on structural abnormalities. This discovery suggests that the interplay between the development of the external ear and the heart may involve not only anatomical aspects but also profound implications for the electrophysiological dynamics of the heart. The presence of ECG abnormalities in individuals with microtia goes beyond the realm of structural defects, hinting at the possibility of inherent congenital electrophysiological and conduction abnormalities^24^. This signifies that the intricate orchestration of cellular and molecular processes during embryonic development, which gives rise to both the ear and the heart, may have a more interconnected and nuanced impact than previously recognized.

While the precise mechanisms remain uncertain, a compelling hypothesis draws attention to the pivotal role played by neural crest cell migration processes—an intricate series of events integral to the formation of both the external ear and the heart. Neural crest cells, a unique and versatile group of cells, embark on a transformative journey during early embryonic development. These cells migrate extensively throughout the developing embryo, contributing to the formation of diverse structures, including both the auricular and cardiac tissues^25–27^. An interruption or misregulation in the migration and differentiation of these cells might lead to cascading effects, potentially contributing to the observed cardiac abnormalities in individuals with microtia. Therefore, further investigation to unravel the complexities of neural crest cell dynamics in the context of microtia combined with cardiac irregularity is needed. Understanding how these cells navigate and differentiate to form both the external ear and the heart could shed light on the underlying factors driving the increased prevalence of ECG abnormalities. By elucidating the intricate interplay between neural crest cell migration and differentiation, we may uncover novel insights into the shared developmental origins of auricular and cardiac structures, paving the way for a deeper comprehension of congenital anomalies associated with microtia.

Several limitations existing in the present study should be acknowledged. First, this study at a single medical center might have data migration of population that affected the evaluation. Second, The absence of a matched control group of complete healthy individuals which could allow for a more rigorous comparison. Third, the cohort of individuals with microtia in our study exhibits clinical heterogeneity in terms of the severity of ear anomalies and associated cardiac abnormalities. Subgroup analyses based on specific phenotypic characteristics were limited by the retrospective nature of the present study, which could not capture the complete relevant ear photos for classification.

## Conclusion

Our study has demonstrated the characteristics and incidence of ECG abnormalities in patients with microtia. And the occurrence of ECG abnormalities in microtia patients was higher than that in the control group. Furthermore, these findings underscore the potential congenial defect on cardiac electrophysiology beyond the presence of CHD, which coincide with microtia. For plastic surgeons and anesthesiologists, it is essential to pay attention to ECG in the cardiac assessment to ensure the safety of the peri-operative period.

## Data Availability

Data herein reported are fully available in Tables 1, 2, 3 and Figs. 1, 2, 3, 4.
